# Platelet FcγRIIA is dispensable for hemostasis and arterial thrombosis in the absence of FcγRIIA-specific extracellular stimuli

**DOI:** 10.1016/j.rpth.2026.106853

**Published:** 2026-07-14

**Authors:** Florian Tupin, Stephanie Magnenat, Clarisse Mouriaux, Pierre H. Mangin, Béatrice Hechler

**Affiliations:** Université de Strasbourg, Établissement Français du Sang (EFS) Grand Est, Institut National de la Santé et de la Recherche Médicale (INSERM) Unité Mixte de Recherche (UMR)_S1255 department, Fédération de Médecine Translationnelle de Strasbourg (FMTS), Strasbourg, France

**Keywords:** animal model, arterial thrombosis, FcγRIIA, hemostasis, platelets

## Abstract

**Background:**

FcγRIIA, a high-affinity receptor for immunoglobulin G immune complexes with no ortholog in mice, has a prominent role in host defense against pathogens via immune cell activation. A role for platelet FcγRIIA in enhancing thrombosis was suggested, involving interplay between α_IIb_β_3_ integrin and FcγRIIA, without requiring FcγRIIA-specific extracellular stimuli. Recent data challenged this view, showing that FcγRIIA inhibition neither impaired platelet spreading on fibrinogen nor prevented thrombus growth and stability under flow.

**Objectives:**

This study assessed contribution of FcγRIIA to platelet function and experimental hemostasis and thrombosis without FcγRIIA-specific trigger.

**Methods:**

The role of FcγRIIA in platelet functions was evaluated using washed human platelets following blockade of FcγRIIA with the monoclonal antibody IV.3, and transgenic mice expressing human FcγRIIA (FcγRIIA^+^) when compared with wild-type (WT) mice. The importance of FcγRIIA in hemostasis and arterial thrombosis was assessed using a tail vein transection bleeding model and various thrombosis models with real-time intravital microscopy, respectively.

**Results:**

Aggregation of washed human platelets to various agonists was identical in the absence or presence of IV.3, as was aggregation of washed WT and FcγRIIA^+^ platelets. Adhesive and aggregation properties of FcγRIIA^+^ and WT platelets were identical when perfused over collagen under flow. FcγRIIA^+^ and WT mice exhibited similar tail bleeding time and kinetic and extent of thrombus formation in various arterial thrombosis models.

**Conclusion:**

FcγRIIA does not contribute to platelet functions during hemostasis and arterial thrombosis in the absence of FcγRIIA-specific extracellular stimulation, which does not support a role for FcγRIIA in amplifying outside-in α_IIb_β_3_ signaling.

## Essentials

• The high-affinity receptor for IgG immune complexes FcγRIIA is key in immunothrombosis.
• We questioned FcγRIIA’s role in hemostasis and thrombosis without FcγRIIA extracellular trigger.
• FcγRIIA does not amplify platelet aggregation and *in vitro* thrombosis.
• FcγRIIA is dispensable for hemostasis and thrombosis without FcγRIIA extracellular stimuli.

## 1 Introduction

FcγRIIA or CD32A is a high-affinity receptor for immunoglobulin (Ig) G immune complexes, expressed on human platelets and myeloid immune cells, whereas no corresponding ortholog is present in mouse [1]. Transgenic mice expressing human FcγRIIA/CD32A (FcγRIIA^+^ mice) recapitulate the corresponding human expression patterns and have been shown to reliably mimic several human physiological and pathological responses [1,2]. FcγRIIA plays an important role in immunity allowing release of proinflammatory and antimicrobial molecules by granulocytes, monocytes, or platelets [[3], [4], [5], [6], [7]]. It also promotes neutrophil phagocytosis [8]—elimination of virus-infected cells by monocytes and granulocytes [9]—and contributes to the clearance of immune complexes [10,11]. FcγRIIA is implicated in various immune disorders such as rheumatoid arthritis [12], vasculitis [13], glomerulonephritis [14], lupus [15], anaphylaxis [16,17], and immune transfusion–related acute lung injury [18]. Platelet FcγRIIA is a major contributor of lupus, IgG-dependent anaphylaxis, and immune transfusion–related acute lung injury pathophysiology [15,16,18] and is key in thrombotic and thrombocytopenic complications of heparin immune thrombocytopenia [19,20], vaccine-induced immune thrombotic thrombocytopenia, and thrombosis in patients with COVID-19 [21,22].

FcγRIIA is composed of 2 extracellular Ig-like domains and 1 intracellular Immune Tyrosine-based Activation Motif (ITAM) with 2 phosphorylable tyrosines [23]. On platelets, crosslinking of FcγRIIA by IgG engagement causes phosphorylation of the ITAM tyrosines by sarcoma tyrosine kinase (Src) such as Fyn, Lck, and Lyn. Spleen tyrosine kinase (Syk) is then recruited, followed by the downstream activation of numerous proteins including the linker for activation of T cell protein, Slp76 protein, and phosphoinositide 3-kinase, which allow the generation of phosphatidylinositol-3,4,5-triphosphate. This phospholipid forms a membrane anchor site for phospholipase Cγ2 recruitment and activation, leading to the formation of diacylglycerol and inositol triphosphate, followed by an increase in the intracellular calcium concentration. This leads to granule secretion, thromboxane A2 synthesis resulting in α_IIb_β_3_ integrin activation, and platelet aggregation [24,25].

It has been suggested that platelet FcγRIIA could enhance outside-in signal mediated by α_IIb_β_3_ integrin, without requiring FcγRIIA-specific extracellular stimulation [26,27]. Indeed, thrombin receptor agonist peptide (TRAP-6)–induced binding of soluble fibrinogen to washed human platelets or direct platelet binding to immobilized fibrinogen resulted in tyrosine phosphorylation of FcγRIIA and its downstream effectors Syk and phospholipase Cγ2, which were strongly reduced in the presence of the anti–FcγRIIA-specific monoclonal antibody (mAb) IV.3 [26]. Human platelet spreading on fibrinogen was also shown to be markedly reduced in the presence of IV.3 and in platelets derived from a patient with ∼60% reduced expression of FcγRIIA [26]. This functional interplay between α_IIb_β_3_ and FcγRIIA led to enhanced platelet aggregation to various agonists and enhanced thrombus formation on a collagen-coated surface under flow conditions [[26], [27], [28]]. This amplification effect was confirmed in transgenic mice expressing human FcγRIIA and resulted in enhanced thrombus formation in both arteries and veins [27].

Interestingly, we recently reported that IV.3 did not inhibit adhesion and spreading of human platelets on fibrinogen, nor prevent thrombus growth and stability on a collagen-coated surface under flow conditions [29], challenging the role of FcγRIIA in amplifying α_IIb_β_3_ functions and the importance of FcγRIIA in hemostasis and thrombosis in the absence of FcγRIIA-specific extracellular trigger, as raised recently [30,31]. Thus, the aim of the present study was to revisit in depth the contribution of FcγRIIA in platelet function and in experimental models of hemostasis and arterial thrombosis in the absence of FcγRIIA-specific extracellular stimulation.

## 2 Methods

### 2.1 Reagents

Xylazine (Rompun) and ketamine (Imalgene 1000) were from Bayer and Merial, respectively. Isoflurane was purchased from Virbac. The complete mouse antihuman FcγRIIa monoclonal antibody (IV.3) was from Stemcell Technologies. The IV.3 Fab fragment was provided by Dr Friederike Jönsson (Institut Pasteur, France). The donkey antimouse F(ab’)_2_ was from Jackson ImmunoResearch. Fibrinogen came from an in-house preparation, and apyrase was purified as previously described [32]. The prostaglandin (PG)I_2_ and the protease-activated receptor 4 agonist peptide (AYPGKF-NH_2_) were purchased from Sigma. The TRAP-6 was from PolyPeptide, adenosine 5’-diphosphate (ADP) was from MAST DIAGNOSTIC, and U46619 from Calbiochem. The mouse antihuman CD9 monoclonal antibody (ALMA.1) was homemade. Horm fibrillar type I collagen from equine Achilles tendon was purchased from Takeda. Collagen-related peptide (CRP) came from CambCol. The cell-permeant green-fluorescent lipophilic dye DIOC_6_ (3,3′-dihexyloxacarbocyanine iodide) was from Molecular Probes, ferric chloride (FeCl_3_) from Prolabo, acid citrate dextrose (ACD) from Macopharma, and recombinant hirudin from Transgene.

### 2.2 Mice

Mice of C57BL/6J genetic background were used at adult age, ranging from 7 to 12 weeks except for the laser-induced mesenteric arteriole thrombosis model in which mice were used at 3 weeks. Transgenic mice hemizygous for the gene encoding the human FcγRIIA receptor (FcγRIIA^+^) [2] were provided by P. Bruhns (Institut Pasteur, Paris, France) and bred with wild-type (WT) mice at the animal facility of Établissement Français du Sang Grand Est (approval no. G-67-482-10) to generate WT and hemizygous FcγRIIA^+^ littermates. This breeding strategy ensured the maintenance of a consistent genetic background and minimized drift over generations. In FcγRIIA^+^ mice, FcγRIIA is expressed in all myeloid cells, at levels similar to those found in humans [1]. The presence of the hCD32A transgene was assessed by polymerase chain reaction analysis of genomic DNA using the following primers: 5'-CAATTTTGCTGCTATGGGC-3' and, 5'-CTGGTCAAGGTCACATTCTTC-3'. Experiments were performed on FcγRIIA^+^ mice and their WT littermates. Mice were housed in standard individually ventilated cages and were maintained on standard rodent chow. All animal experimentation procedures were validated by the Regional Committee of Ethics in Animal Experimentation of Strasbourg (C.R.E.M.E.A.S; CEEA-35) and were authorized by the Ministry of Higher Education, Research and Innovation, in accordance with European directives (APAFIS#22083-2019092013222943).

### 2.3 Blood collection

Blood samples from healthy donors were obtained from the French National Blood Donor Service (Établissement Français du Sang Grand Est) with the informed consent of the donors. Blood was drawn into ACD (1:6) as the anticoagulant. Mice were anesthetized with xylazine (20 mg/kg body weight) and ketamine (100 mg/kg body weight) and maintained at 37 °C. Mouse blood was collected from the abdominal aorta, using a 25-gauge needle into ACD (1:6) for preparation of washed platelets or into hirudin (100 U/mL) for flow-based assays.

### 2.4 Platelet washing procedure

Platelets were isolated from ACD-anticoagulated blood as previously described [32]. Briefly, platelets were separated from other blood component by differential centrifugation and washed twice at 37 °C in Tyrode buffer (137 mM NaCl, 2 mM KCl, 12 mM NaHCO_3_, 0.3 mM NaH_2_PO_4_, 1 mM MgCl_2_, 2 mM CaCl_2_, 5.5 mM glucose, 5 mM HEPES, pH 7.3, 295 mOsm/L) containing 0.35% purified human serum albumin (HSA) and 0.5 μM PGI_2_. Finally, platelets were suspended in Tyrode buffer containing 0.35% HSA and 0.02 U/mL of the adenine nucleotide scavenger apyrase, a concentration sufficient to prevent desensitization of platelet responses to ADP [33], and no PGI_2_. The final suspension was adjusted to 3 × 10^5^ platelets/μL and were kept at 37 °C throughout all experiments.

### 2.5 Aggregation assays

Aggregation of human or mouse washed platelet suspension was measured at 37 °C for 5 minutes by a turbidimetric method in an APACT 4004 aggregometer. A 270-μL aliquot of platelet suspension (3.0 × 10^5^ platelets/μL) was stirred at 1100 rpm in the presence of fibrinogen (250 μg/mL; except for stimulation with thrombin) and activated by addition of 30 μL of the indicated agonist. Full IgG or Fab fragment of IV.3 mAb were added 30 seconds prior the addition of the agonist.

### 2.6 *In vitro* flow–based assay

Microfluidic channels (0.09 mm/1 mm) were coated for 1 hour at room temperature with collagen (200 μg/mL) and blocked with phosphate-buffered saline containing 1% fatty acid–free HSA for 30 minutes at room temperature. Hirudinated (100 U/mL) whole blood from WT or FcγRIIA^+^ mice were perfused through the coated chambers with a syringe pump (Harvard Apparatus) at 37 °C at a wall shear rate of 1500/s for 3 minutes. Thrombus formation on collagen was monitored by labeling the platelets with DIOC_6_ (1 μM) for 10 minutes at 37 °C. After excitation with a 488-nm argon-ion laser, thrombi were detected by measuring the fluorescence in the range 493 to 575 nm using a confocal Leica SP8 inverted microscope with a resonant scanner and a 40× oil objective. Series of optical sections in xyz from the base to the peak of the thrombi were recorded over time. The images were stacked and analyzed with ImageJ software (NIH) to determine the thrombus volume.

### 2.7 Tail vein transection bleeding model

Mice were anesthetized with isoflurane (Vetflurane) and maintained at 37°C. The tail bleeding time was measured as previously reported [34]. The time required for arrest of bleeding, number of rebleeds, and the volume of blood loss were determined over a 30-minute period.

### 2.8 Carotid artery thrombosis induced by FeCl_3_ injury

Mice (8-10 weeks old) were anesthetized with an intraperitoneal injection of ketamine (100 mg/kg) and xylazine (10 mg/kg) and maintained at 37 °C. They received an intravenous injection of the fluorescent dye, DIOC_6_ (5 μL of a 100 μM solution/g of body weight) to label platelets and assist thrombus visualization. The common carotid arteries were exposed, and vascular injuries were induced by lateral external application of a Whatman filter paper (3 × 1 mm) saturated with 7.5% FeCl_3_ for 2.5 minutes. Thrombus formation was monitored in real time for 18 minutes under a fluorescent microscope (Leica Z16 APO; Leica Microsystems SA) coupled to a charge-coupled device camera (CoolSNAP HQ2; Photometrics). Fluorescent images were acquired sequentially (1 image/5 s) and analyzed with Metamorph software.

### 2.9 Abdominal aortic thrombosis induced by mechanical injury

Mice were anesthetized with a mixture of ketamine (100 mg/kg) and xylazine (10 mg/kg) and injected with DIOC_6_ (5 μL of a 100 μM solution/g of body weight) to label platelets. The abdominal aorta was exposed, and mechanical injury was induced by pinching the aorta with hemostatic forceps for 15 seconds as described [35]. Thrombus formation was monitored in real time for 20 minutes using a fluorescence macroscope (Leica Z16 APO) coupled to a charge-coupled device camera (CoolSNAP HQ2; Photometrics). Fluorescent images were acquired sequentially (1 image/20 s) and analyzed with ImageJ software (National Institutes for Health).

### 2.10 Mesenteric arterioles thrombosis induced by laser injury

Three-week-old male and female mice weighing 12 to 15 g were anesthetized with an intraperitoneal injection of ketamine (100 mg/kg) and xylazine (10 mg/kg) and maintained at 37 °C. The use of young mice is necessary to minimize fat surrounding the vessels, facilitating focusing of the laser beam and visualization of the thrombus. Platelets were labeled by DIOC_6_ (5 μL of a 100 μM solution/g of body weight) injection into the jugular vein. After exposure of their mesentery, a localized injury of an arteriole 65 to 100 μm in diameter was induced with a 440-nm pulsed nitrogen dye laser applied through the objective (40×) of an inverted Leica DMIRB microscope with a Micropoint laser system (Photonics Instruments). For superficial injury, laser intensity was 18 on a 0 to 26 scale. The laser beam was focused on the same area for 15 to 20 seconds on the luminal face of the vessel to cause detachment of a wall fragment under transmitted light. A 10 pulses/s, 150 to 200 laser hits were administered. The beam was then displaced 60 to 70 μm downstream along the vessel wall in a 2- to 3-second interval. For the deep injury, laser intensity was set at 22, and the beam was focused for 30 to 40 seconds (300-400 hits) to cause sudden rupture and formation of a crater inside the vessel wall. For each type of lesion, 2 to 5 arterioles per animal were targeted over a period of 1 hour with only 1 injury per vessel. Thrombus formation was monitored in real time by brightfield and fluorescence microscopy using a CMOS ORCA Flash V2 camera (Hamamatsu Photonics). Images were acquired with Metamorph and analyzed with SlideBook software (Intelligent Imaging Innovations).

### 2.11 Statistical analysis

**Figure 1:**
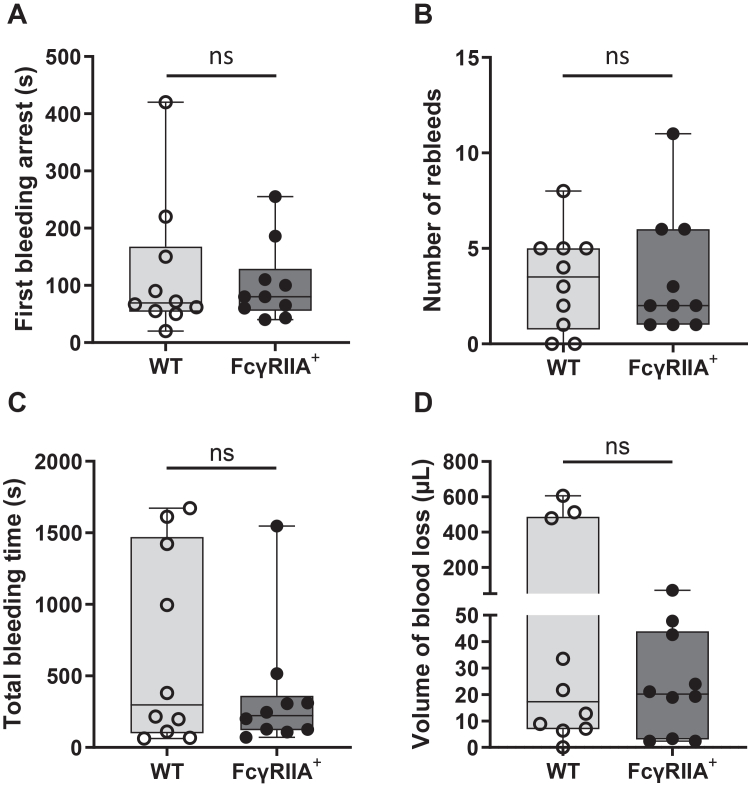
FcγRIIA does not contribute to hemostasis in mice. (A–D) A 3-mm segment from wild-type (WT) and FcγRIIA^+^ mouse tails were transversally severed, and 4 parameters were evaluated over 30 minutes. (A) The time required for the first arrest of bleeding (Mann–Whitney U-test, *P* = .90; *n* = 10), (B) the number of rebleeds (Mann–Whitney U-test, *P* = .98; *n* = 10), (C) the total bleeding time (Mann–Whitney U-test, *P* = .64; *n* = 10), and (D) the volume of blood lost (Mann–Whitney U-test, *P* = .74; *n* = 10).

**Figure 2:**
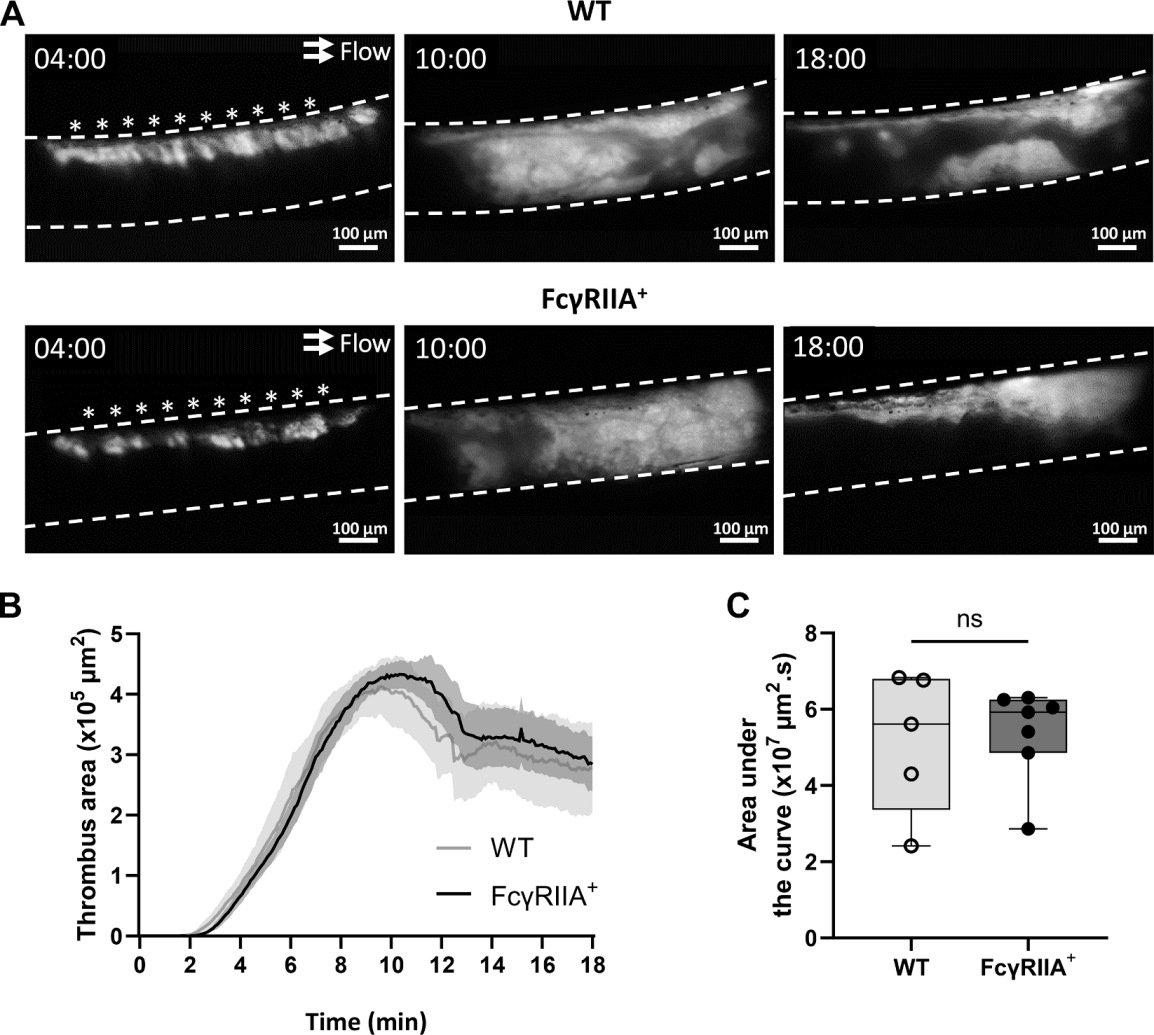
FcγRIIA does not aggravate thrombosis of carotid artery induced by FeCl_3_ injury in mice. (A–C) Thrombosis was triggered in wild-type (WT; *n* = 5) and FcγRIIA^+^ (*n* = 7) mice by application of a filter paper saturated with 7.5% FeCl_3_ for 2.5 minutes to the common carotid artery. (A) Representative fluorescence images of the thrombus at the indicated time points after injury. Scale bar: 100 μm. Arrows indicate the direction of blood flow. Asterisks indicate the contact site with FeCl_3_ and the dotted lines the borders of the vessels. (B) Time course of thrombus growth represented by its surface area (mean values ± SEM). (C) Area under the curve of the thrombus (Mann–Whitney U-test, *P* > .99).

**Figure 3:**
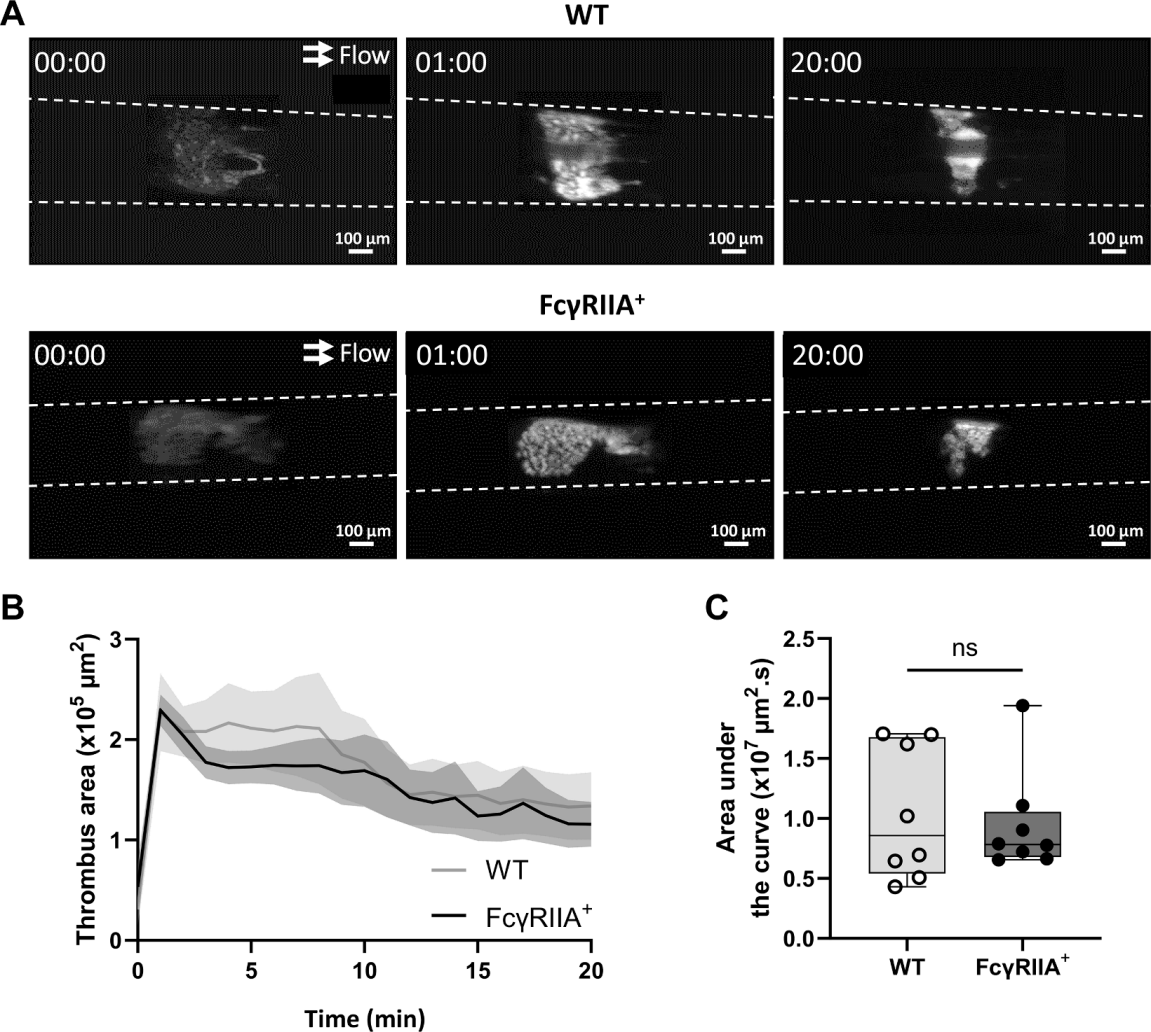
FcγRIIA receptor does not aggravate thrombosis of abdominal aorta induced by forceps injury in mice. (A–C) Thrombosis was triggered in wild-type (WT) and FcγRIIA^+^ mice by compression of the abdominal aorta with forceps. (A) Representative fluorescence images of the thrombus at the indicated time points after injury. Scale bar: 100 μm. Arrows indicate the direction of blood flow and the dotted lines the borders of the vessels. (B) Time course of thrombus growth represented by its surface area (mean values ± SEM; *n* = 8 mice). (C) Area under the curve of the thrombus (Mann–Whitney U-test, *P* = .80; *n* = 8 mice).

**Figure 4:**
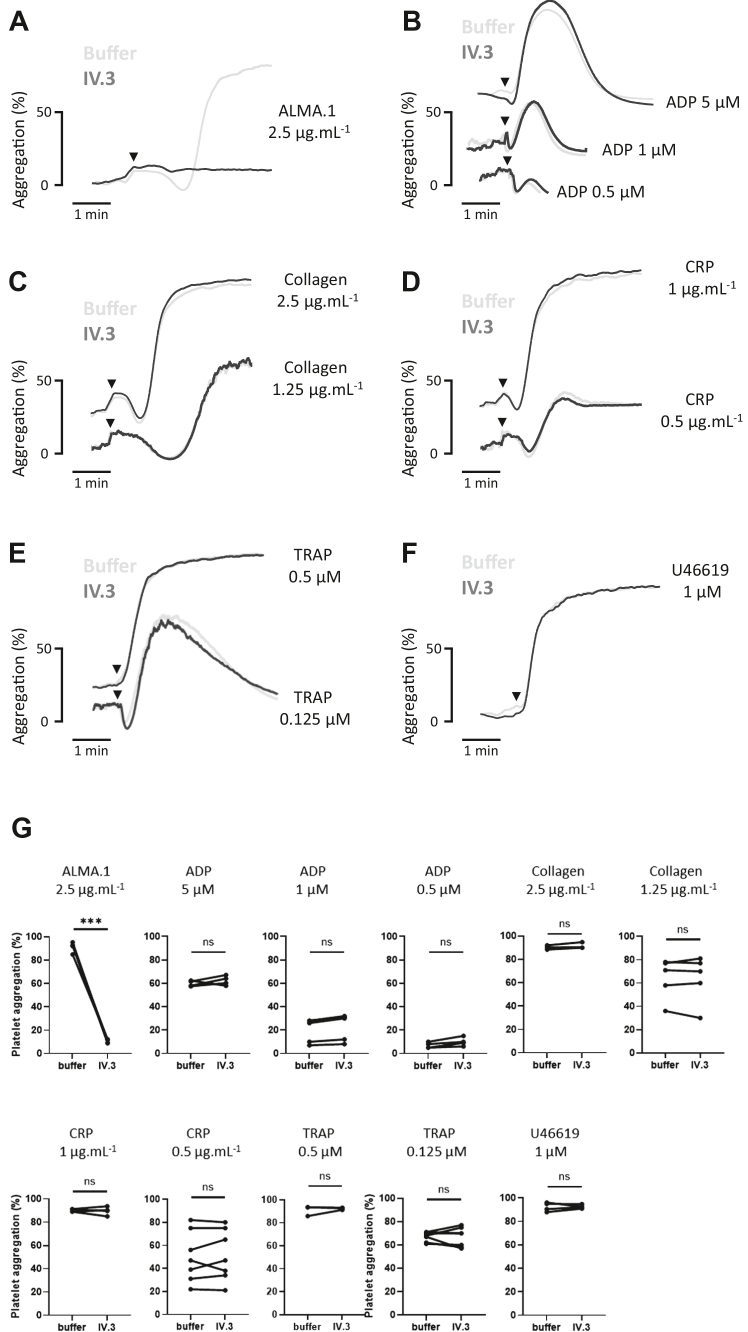
FcγRIIA does not contribute to aggregation of washed human platelets in the absence of FcγRIIA-specific extracellular stimulation. (A–G) Washed platelets (3.0 × 10^5^ platelets/μL) from healthy donors in the absence or presence of the mouse antihuman FcγRIIA monoclonal antibody IV.3 (10 μg/mL), were stimulated with (A) mouse antihuman CD9 monoclonal antibody (ALMA.1; 2.5 μg/mL), (B) adenosine diphosphate (ADP; 5, 1, and 0.5 μM), (C) collagen (2.5 and 1.25 μg/mL), (D) collagen-related peptide (CRP; 1 and 0.5 μg/mL), (E) thrombin receptor activator peptide (TRAP; 0.5 and 0.125 μM), (F) thromboxane A2 analog (U46619 1 μM). Arrows represent the addition of agonist. Data are from 1 experiment representative of minimum 3 independent experiments giving identical results. (G) Donor-specific pairwise comparison of maximum platelet aggregation (2-tailed paired *t*-test or Wilcoxon matched-pairs test, ∗∗∗*P* < .001; ns, *P* > .05; *n* = 3-7).

Statistical analyses were performed with GraphPad software (Prism 10.3.1). The normal distribution of the data was determined using the Shapiro–Wilk normality test. When data followed normal distribution, they were represented in bar graph as the mean ± SEM and analyzed using 2-tailed unpaired *t*-test. When data did not follow normal distribution, they were represented as box-and-whisker plots showing the median (minimum; maximum) and the IQR (25th-75th percentiles) and were analyzed using the Mann–Whitney U-test (Figures 1A–D, 2C, and 3C). Specifically for Figure 4G, donor-specific pairwise comparison was represented as dot plot with connecting lines for paired samples and were analyzed using 2-tailed paired *t*-test or Wilcoxon matched-pairs test when normality was not met. A *P* value of ≤ .05 was considered statistically significant.

## 3 Results

### 3.1 Assessment of FcγRIIA contribution to platelet function in the absence of FcγRIIA-specific extracellular stimulation

We first assessed the number of copies of FcγRIIA per platelet, a key determinant of reactivity and inhibition. Evaluation of a subset of 6 healthy donors indicated an average of 1579 ± 188 receptors/platelet (Supplementary Figure 1), within the previously reported range [36,37]. Activation of human platelets by IgG antibodies usually depends on their binding both to the target antigen such as the anti-CD9 mAb ALMA.1 and to FcγRIIA [38]. Thus, ALMA.1 (2.5 μg/mL) induced potent aggregation of platelets isolated from healthy donors, which was prevented by the specific anti-FcγRIIA function–blocking mAb IV.3 (10 μg/mL) (Figure 4A,G), showing functionality of this receptor. To examine the potential contribution of FcγRIIA to platelet aggregation, we evaluated the effect of IV.3 on washed platelet aggregation induced by weak and strong agonists. Aggregation induced by ADP (5, 1, and 0.5 μM), collagen (2.5 and 1.25 μg/mL), CRP (1 and 0.5 μg/mL), TRAP-6 (0.5 and 0.125 μM), or U46619 (1 μM) was similar in the absence or presence of IV.3 full IgG (Figure 4B–G) or Fab fragment (Supplementary Figure 2), suggesting that FcγRIIA does not play a major role in platelet aggregation in the absence of FcγRIIA-specific extracellular stimulation.

**Figure 5:**
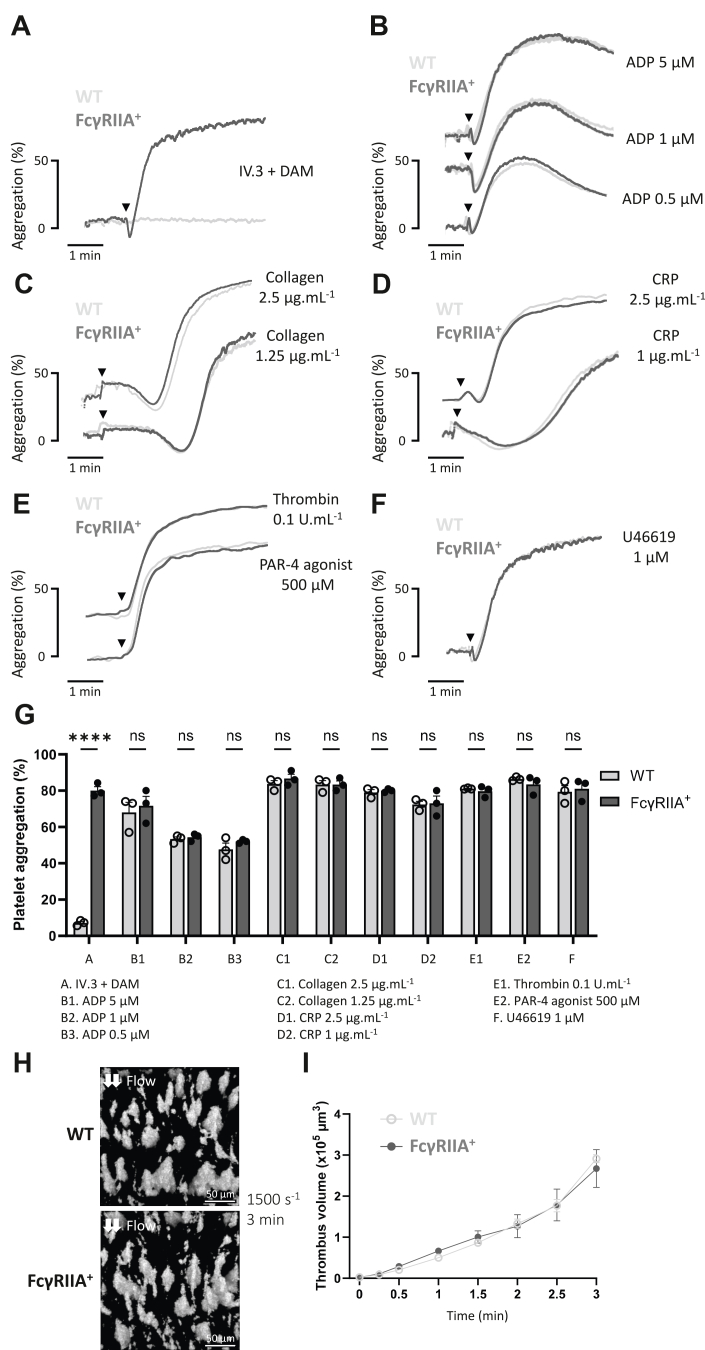
FcγRIIA does not exacerbate mouse platelet aggregation in the absence of FcγRIIA-specific extracellular stimulation. (A–H) Washed platelets (3.0 × 10^5^ platelets/μL) from wild-type (WT) or FcγRIIA^+^ mice were stimulated with (A) IgG immune complexes composed of mouse antihuman FcγRIIA monoclonal antibody (IV.3; 1 μg/mL) and a donkey antimouse F(ab’)_2_ (DAM; 15 μg/mL), (B) ADP (5, 1, and 0.5 μM), (C) collagen (2.5 and 1.25 μg/mL), (D) collagen-related peptide (CRP; 2.5 and 1 μg/mL), (E) thrombin (0.1 U/mL), protease-activated receptor 4 agonist (500 μM), and (F) thromboxane A2 analog (U46619 1 μM). Arrows represent the addition of agonist. Data are from 1 experiment representative of 3 independent experiments giving identical results. (G) Maximum wild-type (WT) or FcγRIIA^+^ platelet aggregation to the various agonists (unpaired *t*-test, ∗∗∗∗*P* < .0001; ns, *P* > .05; *n* = 3). (H, I) Hirudinated whole blood from WT or FcγRIIA^+^ mice (*n* = 4) was perfused over a collagen-coated surface at 1500/s for 3 minutes. (H) Representative confocal Z stack images of thrombi at 3 minutes. Scale bar: 50 μm. Arrows indicate the direction of blood flow. (I) Time course of thrombus growth represented by its volume (mean values ± SEM; unpaired *t*-test at 3 minutes, *P* = .48; *n* = 4).

To further explore the importance of FcγRIIA in platelet aggregation to a series of activators, we compared aggregation responses of washed platelets derived from WT and FcγRIIA^+^ mice. FcγRIIA was functional on platelets from FcγRIIA^+^ mice, as FcγRIIA^+^ platelets were able to aggregate, unlike WT platelets, in response to stimulation with IV.3 antibody crosslinked with F(ab’)_2_ fragments of a donkey antimouse secondary antibody (Figure 5A,G). In contrast, ADP (5, 1, and 0.5 μM), collagen (2.5 and 1.25 μg/mL), CRP (2.5 and 1 μg/mL), thrombin (0.1 U/mL), a specific PAR-4 agonist peptide (500 μM), or U46619 (1 μM), promoted similar aggregation of washed platelets from WT and FcγRIIA^+^ (Figure 5B–G). Next, the contribution of FcγRIIA to thrombus formation under flow conditions was tested by perfusion of hirudinated whole blood from WT or FcγRIIA^+^ mice into microfluidic chambers coated with collagen at a wall shear rate of 1500/s. Kinetic of thrombus formation was similar between FcγRIIA^+^ and WT mice during 3 minutes of blood perfusion (Figure 5H,I). Overall, these results indicate that FcγRIIA does not amplify platelet function in the absence of FcγRIIA-specific extracellular stimulation.

### 3.2 Assessment of FcγRIIA contribution to hemostasis in mice in the absence of FcγRIIA-specific extracellular stimulation

To assess whether FcγRIIA contributed to hemostasis, we evaluated the bleeding after calibrated tail transection in WT and FcγRIIA^+^ mice, using the following 4 parameters: the first time of bleeding arrest, the number of rebleeds, the total bleeding time, and the volume of blood loss. The time of first bleeding arrest was not significantly different between WT and FcγRIIA^+^ mice (Figure 1A). This was also the case for the number of rebleeds (Figure 1B). The total bleeding time was also not significantly different between WT and FcγRIIA^+^ mice, as was the total volume of blood loss (Figure 1C,D). Together, these results provide evidence that FcγRIIA is not a central contributor of hemostasis in mice.

### 3.3 Assessment of FcγRIIA contribution to arterial thrombosis in mice

**Figure 6:**
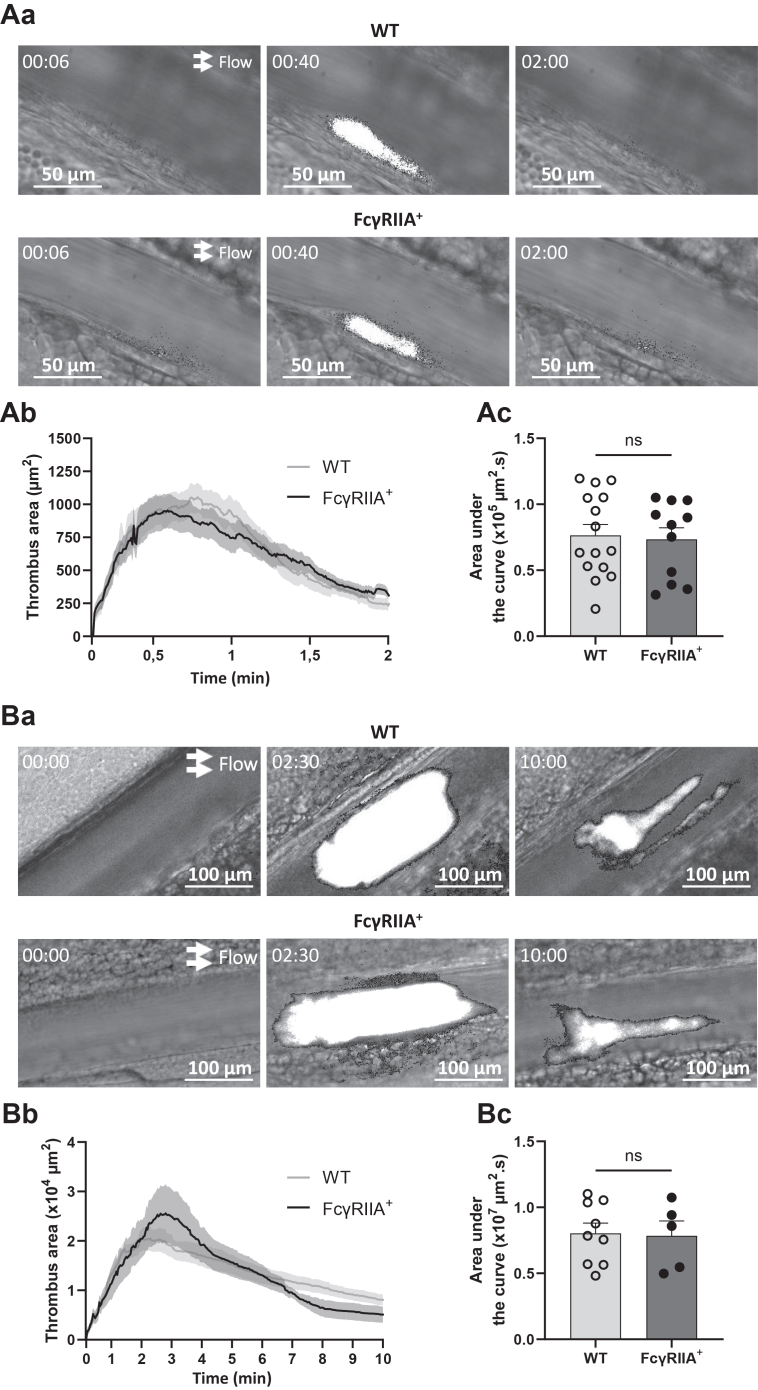
FcγRIIA does not aggravate thrombosis of mesenteric arterioles induced by laser injury in mice. (A,B) Thrombosis was triggered in wild-type (WT) and FcγRIIA^+^ mice by superficial (A) or deep (B) laser injury of a mesenteric arteriole. Arrows indicate the direction of blood flow. (A) Superficial laser injury. (Aa) Representative images of the thrombus at the indicated time points after injury. Scale bar: 50 μm. (Ab) Time course of thrombus growth represented by its surface area (mean values ± SEM; *n* = 15 vessels in 7 WT mice, *n* = 11 vessels in 5 FcγRIIA^+^ mice). (Ac) Area under the curve of the thrombus (unpaired *t*-test, *P* = .80). (B) Deep laser injury. (Ba) Representative images of the thrombus at the indicated time points after injury. Scale bar: 100 μm. (Bb) Time course of thrombus growth represented by its surface area (mean values ± SEM; *n* = 9 vessels in 7 WT mice; *n* = 5 vessels in 5 FcγRIIA^+^ mice). (Bc) Area under the curve of the thrombus (unpaired *t*-test, *P* = .88).

We evaluated whether FcγRIIA contributed to arterial thrombus formation using various models that are not experimentally triggered by FcγRIIA-specific extracellular stimulation. Various experimental models of arterial thrombosis were used, differing in the nature of the vessel and local rheology, the method to induce injury, and the contribution of thrombin to thrombosis. We previously established a model of laser-induced thrombosis in mesenteric arterioles with 2 levels of injury (superficial and deep) producing a transient thrombus or a larger subocclusive thrombus, respectively, with only deep injuries being sensitive to thrombin blockade [39,40]. Upon superficial or deep laser injuries of mesenteric arterioles, the kinetics of thrombus formation monitored by real-time intravital imaging were similar between FcγRIIA^+^ and WT mice (Figure 6Aa,b,Ba,b). In agreement, area under the curve (AUC) was not statistically different between WT and FcγRIIA^+^ mice (Figure 6Ac,Bc). Similarly, FeCl_3_-induced injury of the carotid artery did not impact the kinetic of thrombus formation between FcγRIIA^+^ and WT mice (Figure 2A,B), with AUC presenting no statistical difference (Figure 2C). Finally, mechanical injury of the abdominal aorta also resulted in a similar kinetic of thrombus formation (Figure 3A,B), without any difference in AUC between WT and FcγRIIA^+^ mice (Figure 3C). Overall, these results provide evidence that, in the absence of FcγRIIA-specific extracellular stimulation, FcγRIIA does not contribute to thrombus formation in various experimental models of arterial thrombosis.

## 4 Discussion

In this study, we evaluated the contribution of FcγRIIA to the function of human and mouse platelets, by using various *in vitro* and *in vivo* models of hemostasis and arterial thrombosis that are not experimentally driven by FcγRIIA-specific extracellular stimulation. The present results indicate that FcγRIIA neither regulate nor amplify platelet responses in *in vitro* turbidimetric platelet aggregometry and microfluidic assay in the absence of FcγRIIA-specific extracellular triggers. In addition, we show that FcγRIIA does not contribute to hemostasis or arterial thrombus formation, in 3 arterial thrombosis models differing in the nature of the vessel and local rheology, the method to induce injury, and the contribution of thrombin to thrombosis. Overall, these results do not support a major interplay between platelet FcγRIIA and integrin α_IIb_β_3_ and show that FcγRIIA does not amplify platelet function in the absence of FcγRIIA-specific extracellular stimulation.

Our results indicate that FcγRIIA did not function to amplify platelet aggregation induced by various weak and strong agonists in both humans and in mice expressing human FcγRIIA. In addition, platelet adhesion and thrombus formation on a fibrillar collagen–coated surface under shear flow conditions was found to be similar with blood obtained from FcγRIIA^+^ mice compared with WT mice. This is consistent with our previous results indicating that IV.3 did not prevent human platelet thrombus growth and stability on a collagen-coated surface under flow conditions [29]. These results, combined with the fact that IV.3 did not inhibit adhesion and spreading of human platelets on immobilized fibrinogen [29], do not support the contention that FcγRIIA cooperates with α_IIb_β_3_ integrin to amplify platelet function in multiple *in vitro* models. These results are in contradiction with previous studies showing that the ITAM motif of FcγRIIA amplifies α_IIb_β_3_ integrin outside-in signaling, resulting in enhanced aggregation to various agonists, as well as enhanced thrombus formation on a collagen-coated surface under flow conditions [[26], [27], [28]]. This discrepancy is unlikely to be related to differences in experimental design, since the concentrations of CRP, protease-activated receptor 4 agonist, and U46619 used in the turbidimetric assay were similar to those described in previous studies [27,28]. Of note, evidence supporting a role of FcγRIIA in platelet spreading on fibrinogen reported by Boylan et al. [26] relied primarily on platelets from a patient with a 60% reduced surface expression of FcγRIIA, exhibiting a marked reduction in the ability to spread on immobilized fibrinogen when compared with platelets from healthy individuals [26]. However, patient’s platelets also expressed reduced levels of GPVI, due to autoantibody-mediated shedding of the extracellular domain of GPVI [26], a receptor since shown to be essential for platelet spreading on fibrinogen [29,41,42]. Thus, concomitant reduced levels of GPVI were very likely to explain the observed reduced patient’s platelet spreading on fibrinogen in the study by Boylan et al. [26]. This study also reported that platelets from a patient homozygous for a nonsense *ITGB3* variant (p.Arg724Ter) exhibited reduced FcγRIIA phosphorylation together with a marked defect in platelet spreading on fibrinogen. This mutation truncates the β_3_ subunit and removes most of its cytoplasmic tail [43]. Since α_IIb_β_3_ is the principal platelet fibrinogen receptor and its β_3_ cytoplasmic domain is required for outside-in signaling and platelet spreading, the spreading defect cannot be attributed solely to diminished FcγRIIA phosphorylation and is likely confounded by the integrin truncation itself. Consequently, the relative contributions of reduced FcγRIIA phosphorylation vs loss of β_3_ cytoplasmic signaling remain difficult to disentangle in this context.

Consistent with our *in vitro* results, FcγRIIA did not contribute to hemostasis *in vivo*, as shown in the tail vein transection bleeding model in mice. In addition, we show that FcγRIIA did not contribute to thrombus formation in laser-induced thrombosis in mesenteric arterioles following superficial and deep levels of injury. These findings differ from the reported contribution of FcγRIIA in thrombus formation in a model of laser-induced thrombosis in the microcirculation of mouse cremaster muscle [27]. A closer look at the kinetic of thrombus formation showed that thrombus size appeared slightly larger in FcγRIIA^+^ mice than in WT mice only during the last 30 seconds of recording [27]. Although the latter model closely resembles the deep laser injury model of mesenteric arterioles, both models being depend on thrombin generation and tissue factor exposure, differences in the type, size or tissue environment of the targeted vessels, wall shear rates, procedures used to induce laser injury, and potential differences in mouse housing conditions, diet, or microbiome cannot be ruled out. The absence of contribution of platelet FcγRIIA to arterial thrombosis was further established in 2 other mouse models that differ in the targeted vessel (aorta vs carotid artery) and the type of injury (mechanical vs chemical injury with FeCl_3_), generating thrombotic responses with distinct pharmacologic sensitivities [35,40,44]. It is therefore very unlikely that FcγRIIA is a major player of arterial thrombosis in the absence of FcγRIIA-specific extracellular stimulation. Zhi et al. [27] also reported that expression of human FcγRIIA in transgenic mice was associated with enhanced fibrin generation in a model of electrolytic injury of the femoral vein, while platelet accumulation remained comparable with WT mice, the latter observation being consistent with our *in vitro* and *in vivo* results.

This work, nonetheless, has potential limitations, particularly regarding the platelet FcγRIIA expression level. It is presently unknown whether the FcγRIIA expression levels may influence their relative importance in supporting fibrinogen-dependent platelet aggregation. Such possibility would require further investigation, which is, however, beyond the scope of this study. In addition, FcγRIIA expression level was determined in an independent subset of 6 healthy donors, which prevents establishing a specific link between individual expression levels and functional studies.

In conclusion, our study provides evidence that FcγRIIA is not required for optimal platelet function in multiple *in vitro* models and does not represent a major contributor of hemostasis or experimental arterial thrombosis in mice in the absence of FcγRIIA-specific extracellular stimulation. It is thus unlikely that platelet FcγRIIA is a major contributor of α_IIb_β_3_-mediated outside-in integrin signaling.
