# Supplementary material for: Platelet FcγRIIA is dispensable for hemostasis and arterial thrombosis in the absence of FcγRIIA-specific extracellular stimuli
Source: Res Pract Thromb Haemost. 2026 Jul 14;10(5):106853. doi: 10.1016/j.rpth.2026.106853 (PMC13464229; doi:10.1016/j.rpth.2026.106853)
Supplement: Supplementary file 1 [file mmc1.pdf]

Platelet FcγRIIA is dispensable for hemostasis and arterial thrombosis in the absence of FcγRIIA-specific extracellular stimuli

Florian TUPIN, Stephanie MAGNENAT, Clarisse MOURIAUX, Pierre H. MANGIN, Béatrice HECHLER

Université de Strasbourg, INSERM, Établissement Français du Sang (EFS) Grand Est, UMR_S1255, Fédération de Médecine Translationnelle de Strasbourg (FMTS), Strasbourg, France.

Corresponding Author: Beatrice Hechler Ph.D., Université de Strasbourg, INSERM, Établissement Français du Sang (EFS) Grand Est, UMR_S1255, Fédération de Médecine Translationnelle de Strasbourg (FMTS), Strasbourg, France. Phone: +033 3 88 21 25 25. Email: beatrice.hechler@efs.sante.fr

Data sharing statement: Data can be obtained by writing to Beatrice Hechler.

Supplementary figures:

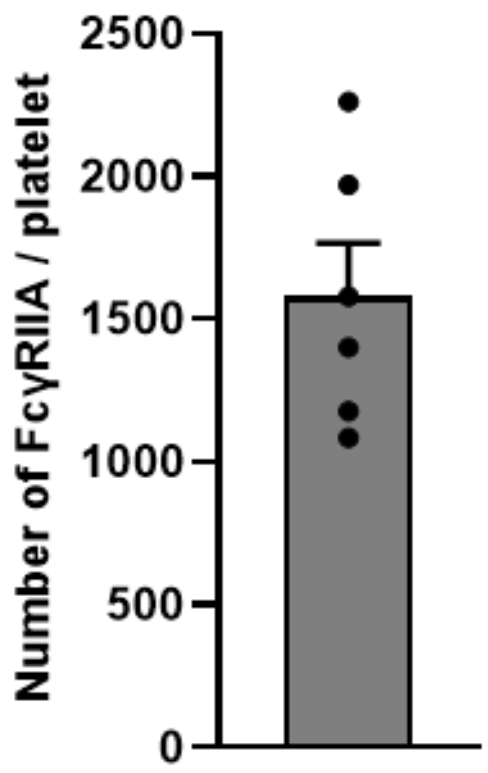

Supplementary Figure S1: Quantification of FcγRIIA copy number on washed human platelets from healthy donors.

The number of surface copies of FcγRIIA per platelet was determined using the mouse anti-human FcγRIIA monoclonal antibody IV.3 (IgG2b) and the Platelet Calibrator Kit (Biocytex). Washed human platelets were preincubated with IV.3 (5 µg.mL^{-1}) for 10 minutes at room temperature and subsequently incubated with a FITC-conjugated anti-mouse IgG for another 10 minutes. Calibrator beads, coated with increasing concentrations of mouse IgG2b antibody molecules (5000; 23 000; 108 000), were stained with FITC-conjugated anti-mouse IgG and analyzed by flow cytometry. The geometric mean fluorescence intensity (MFI) of the 3 bead populations were plotted against the corresponding number of mouse IgG2b molecules. The MFI of IV.3 binding was used to quantify surface expression of FcγRIIA by extrapolation from the linear regression line. FcγRIIA copy number was determined for six blood donors (mean values ± SEM).

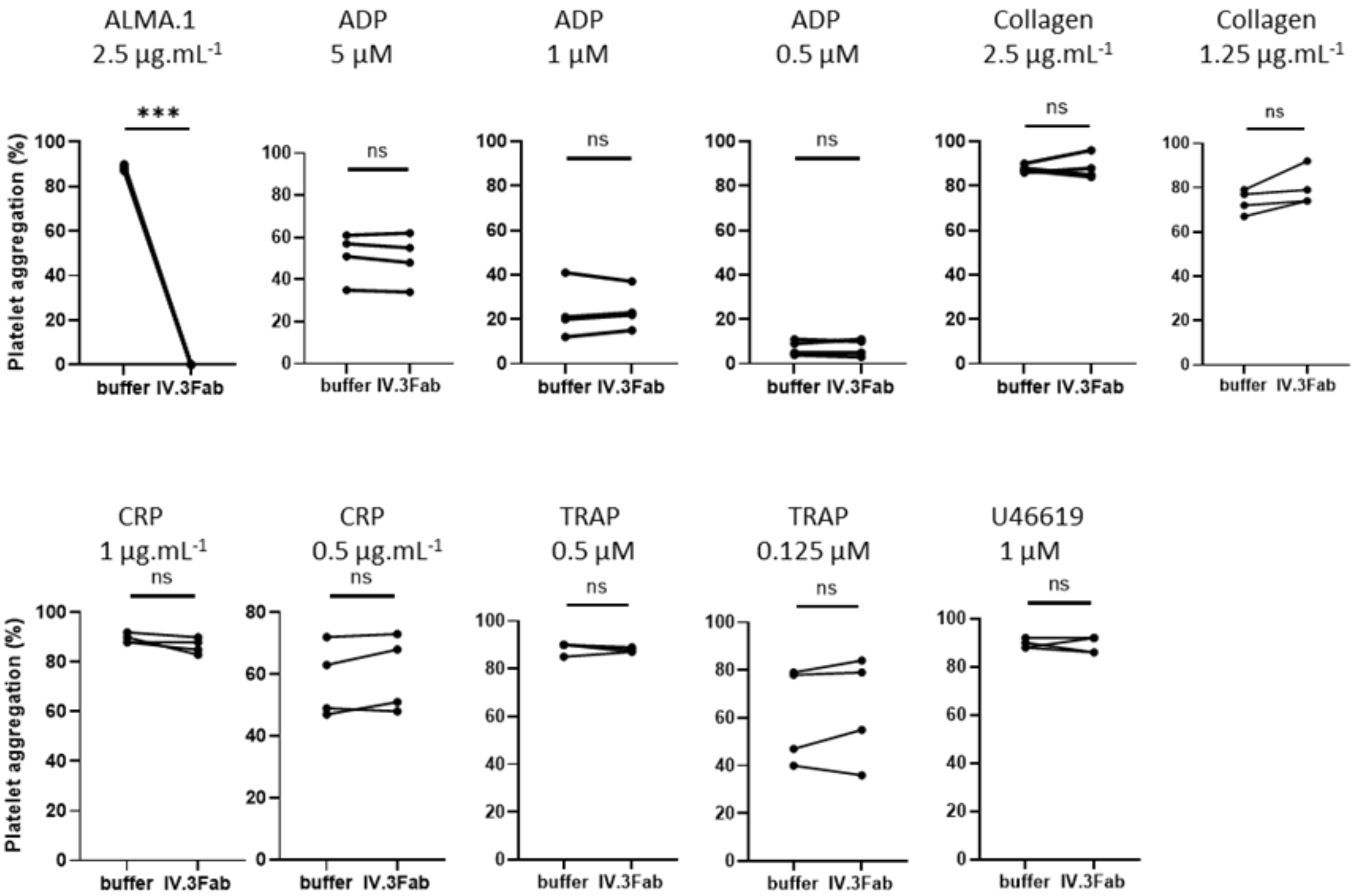

Supplementary Figure S2: Aggregation of washed human platelets in the absence or presence of IV.3 Fab fragment.

Washed platelets (3.0 x 10^5 platelets.µL^{-1}) from healthy donors were stimulated by various agonists in the absence or presence of the Fab fragment from the mouse anti-human FcγRIIA blocking mAb IV.3 (10 µg.mL^{-1}). Data represent donor-specific pairwise comparison of maximum platelet aggregation (two-tailed paired t-test or Wilcoxon matched-pairs test, *** $p<0.001$, ns $p>0.05$, n=4).
